# High-Dose Liraglutide and SGLT2 Inhibitor: A Promising Combination

**DOI:** 10.3390/clinpract12010001

**Published:** 2021-12-21

**Authors:** Marvin Wei Jie Chua

**Affiliations:** Endocrinology Service, Department of General Medicine, Sengkang General Hospital, 110 Sengkang East Way, Singapore 544886, Singapore; marvin.chua.w.j@singhealth.com.sg; Tel.: +65-69302346

**Keywords:** SGLT2 inhibitor, GLP-1 agonist, liraglutide

## Abstract

Sodium-glucose co-transporter-2 (SGLT2) inhibitors and glucagon-like peptide 1 (GLP-1) agonists are important drugs in our armamentarium of treatment for Type 2 diabetes mellitus (DM). In addition to their glucose-lowering effects, they have effects on weight, other metabolic diseases and perhaps most importantly, a cardioprotective and reno-protective effect. Liraglutide is a long-acting GLP-1 agonist which was originally used at 1.8 mg daily for the treatment of DM. However, high-dose liraglutide—liraglutide 3 mg daily, has been demonstrated to be a safe and effective treatment for obesity, with or without DM. In this manuscript, I present two patients who had unusual responses to combination therapy with high-dose liraglutide and SGLT2 inhibitor—marked and/or rapid improvement in glycemic control and weight loss. Drawing from the observations in both cases, I discuss the complementary mechanisms of actions of both drugs, review the clinical effects of combination therapy and distil them into clinical pearls of practical utility for the physician. Given the “clash of the two pandemics” of obesity and COVID-19 and the burgeoning rates of obesity which loom in the near horizon, this is most timely.

## 1. Introduction

Both glucagon-like peptide 1 (GLP-1) agonists and sodium-glucose co-transporter-2 (SGLT2) inhibitors are important drugs in our armamentarium of treatment for Type 2 diabetes mellitus (DM) and obesity. Of the GLP-1 agonists, the clinical utility of high-dose liraglutide (liraglutide 3 mg daily) for the treatment of obesity is of growing interest. This is of particular relevance today, given the “clash of the two pandemics” of obesity and COVID-19 which threaten to lead to an exponential increase in the rates of obesity and its multiple associated complications, not least of which include DM [1]. 

In this report, I describe two patients on combination therapy with high-dose liraglutide and SGLT-2 inhibitor to highlight lesser-known, yet important learning points of practical utility. 

## 2. Case Presentation 

### 2.1. Patient #1

This was a 40-year-old female with obesity as well as young-onset Type 2 DM, diagnosed at the age of 21. There was a family history of DM in her mother, who was diagnosed in her 30 s. There were no prior episodes of diabetic ketoacidosis and insulin auto-antibodies were negative. There was persistent poor glycemic control (HbA1c 12.1–14% since 2012), resulting in microvascular complications of retinopathy and neuropathy. Other comorbidities included hypercholesterolemia on atorvastatin. 

During initial review in July 2019, the patient was obese (weight 95 kg, body mass index (BMI) 39.1 kg/m^2^) and HbA1c was 11.5%. This was despite treatment with premixed insulin 0.79 units/kg/day and metformin 850 mg twice daily, with compliance to all medications. The patient was started on liraglutide, which was up-titrated to 3 mg daily. Three months later, dapagliflozin 10 mg daily was added. The patient was adherent to lifestyle modifications for weight loss and was on a regular exercise regime. The combination of high-dose liraglutide and dapagliflozin led to a rapid and dramatic improvement in glycemic control (decrease in HbA1c of >4%, from 11.5% to 7.2%) after 4 months, which was sustained at 8 months. In contrast, weight loss was gradual—weight was 93 kg in October 2019, representing 1.4% weight loss over 12 weeks, followed by further weight loss to 90 kg in February 2020 and 88 kg in June 2020. The maximum weight loss of 6.3 kg, representing 6.4% of her initial weight, was achieved at 49 weeks following initiation of therapy. Both medications were well-tolerated with no adverse effects. In addition, there was improvement in her lipid profile (LDL cholesterol decreased from 3.07 to 1.71 mmol/L, triglycerides decreased from 2.38 to 1.62 mmol/L and HDL cholesterol increased from 0.84 to 0.98 mmol/L).

### 2.2. Patient #2

This was a 74-year-old female with obesity and multiple associated comorbidities: Type 2 DM, hypertension, hypercholesterolemia, obstructive sleep apnea (OSA), NAFLD and osteoarthritis of the knees.

The patient was first seen in Feb 2019. During initial review, the weight was 101.9 kg and BMI was 40.6 kg/m^2^. Glycemic control was adequate (HbA1c 6.7%) on metformin 1 g twice daily and canagliflozin 100 mg daily. Treatment options were discussed—as the patient was unable to exercise due to knee pain, liraglutide was started for weight control. Six weeks later, the patient had lost 19.8% of weight (weight decreased from 101.9 kg to 81.7 kg, BMI decreased from 40.6 kg/m^2^ to 37.8 kg/m^2^) with improvement in glycemic control (HbA1c decreased from 6.7% to 5.9%). This was achieved with the lowest dose of liraglutide at 0.6 mg daily—despite instructions for up-titration, the patient had intentionally continued low-dose liraglutide due to concerns of adverse effects. Despite subsequent up-titration and continuation of high-dose liraglutide for 12 months, weight plateaued at 81–83 kg, although there was no significant weight regain. There was improvement in OSA as evidenced by decreased apnea hypopnea index (AHI), improvement in lipid profile (LDL cholesterol decreased from 1.73 to 1.64 mmol/L) and maintenance of glycemic control.

### 2.3. Key Clinical Questions

(1) What are the clinical benefits of combination therapy with GLP-1 agonist, specifically high-dose liraglutide, and SGLT2 inhibitor?
(2) Given that Patient #1 experienced a marked improvement in glycemic control while Patient #2 experienced dramatic weight loss—are there any synergistic effects with combination therapy, and what are the underlying mechanisms?
(3) How should combination therapy be initiated—both at the same time or one after the other, and is there any difference in efficacy?
(4) What are the factors which predict response to combination therapy with high-dose liraglutide and SGLT2 inhibitor? How should the physician assess response to treatment?
(5) Do patients on combination therapy experience a plateau in clinical effect, and when does this typically occur?

## 3. Discussion

Does a perfect marriage exist? This is certainly a contentious question, but one that was suggested by Nauck et al. [2] with regard to combination therapy with GLP-1 agonist and SGLT2 inhibitor, and one we shall seek to answer through a closer look at the described patients.

GLP-1 agonists, through an increase in gastrointestinal incretin effect—increase insulin and inhibit glucagon secretion, reduce hepatic glucose production, reduce appetite and decrease muscle-mediated glucose uptake, while SGLT2 inhibitors decrease renal glucose reabsorption. Thus, the combination of GLP-1 agonist and SGLT2 inhibitor corrects seven of the eight key pathophysiological defects of Type 2 DM described in the “Ominous Octet” [3]. This translates to decrease in blood glucose, body weight and blood pressure and beneficial effects on hypertension, hyperlipidemia and non-alcoholic fatty liver disease—benefits clearly observed in the described patients. Perhaps of greatest clinical significance are the cardio-protective and reno-protective effects of both drug classes demonstrated in landmark trials—it remains to be determined whether combination therapy could lead to synergistic benefit in these realms. 

Not only do the two drug classes accentuate each other’s strengths, they also “cover for” each other’s weaknesses. GLP-1 agonists induce satiety and delay gastric emptying, neutralizing appetite stimulation caused by SGLT2 inhibitor-induced glycosuria [3]. GLP-1 agonists increase insulin and suppress glucagon secretion, counteracting SGLT2 inhibitor induced hyperglucagonemia which not only increases endogenous glucose production, but is also implicated in ketone formation [4]. Through this mechanism, GLP-1 agonists might reduce the risk of euglycemic diabetic ketoacidosis, an uncommon but feared complication of SGLT2 inhibitors [5]. As a word of caution, as ketone bodies are theorized to serve as an energy substrate for the failing heart, the clinical impact of decreased ketone availability on cardiovascular outcomes remains to be determined [2]. In addition, SGLT2 inhibitor induced glycosuria might lead to a compensatory increase in hepatic glucose production, which once again is reduced by GLP-1 agonists [6]. This is particularly significant, considering that the degree of hepatic glucose production is proportional to the degree of glycosuria and might offset up to 50% of the glucose excreted in the urine [6]. 

Combination therapy with GLP-1 agonist and SGLT2 inhibitor has been studied in both randomized controlled trials and real-world observational studies, in which liraglutide 0.9–1.8 mg daily, exenatide and dulaglutide were combined with SGLT2 inhibitor [7,8,9,10]. In contrast, both patients in this report received combination therapy with high-dose liraglutide and SGLT2 inhibitor. Although liraglutide was originally used at 1.8 mg daily for the treatment of DM, the clinical utility of high-dose liraglutide for the treatment of obesity, both in patients with and without DM, was first established in the SCALE trials [11,12]. In the SCALE Diabetes study, high-dose liraglutide led to increased reduction in HbA1c and weight as well as decreased use of oral hypoglycemic agents when compared to liraglutide 1.8 mg daily [12]. As far as I am aware, there are no prior studies on the combination of high-dose liraglutide and SGLT2 inhibitor.

There are two methods for initiating combination therapy—sequential initiation and simultaneous initiation. Comparing both methods, simultaneous initiation led to a greater and more rapid reduction in HbA1c and weight, although these differences were unlikely to be significant in the long term [13,14]. The American Diabetes Association (ADA) recommends the sequential initiation of GLP-1 agonist and/or SGLT2 inhibitor in four groups of patients: established atherosclerotic cardiovascular disease, heart failure or chronic kidney disease, obesity and at risk of hypoglycemia. This approach aids compliance and is financially advantageous, given the significant cost of these drugs. Thus, it is likely that sequential initiation would be the more commonly adopted approach, including Patient #1 in whom liraglutide was started followed by SGLT2 inhibitor, and Patient #2 in whom liraglutide was added to existing SGLT2 inhibitor therapy. Gomez et al. [15] found that a greater reduction in HbA1c and weight was observed when GLP-1 agonist was added to existing treatment with an SGLT2 inhibitor, rather than vice versa [13]—which was the approach in Patient #2.

In Patient #1, a marked improvement in glycemic control (decrease in HbA1c of 4.3%) was observed, which is significantly higher than reported in the literature—simultaneous initiation of dapagliflozin and exenatide decreased HbA1c by 1.75%, while addition of empagliflozin to liraglutide 0.9 mg daily and liraglutide 1.8 mg daily to SGLT2 inhibitor decreased HbA1c by just 0.77% and 0.98% respectively [7,9,10]. Given that monotherapy with high-dose liraglutide and dapagliflozin would be expected to lead to HbA1c reduction of 1.3% and 0.4% respectively [12,16], the HbA1c reduction was far greater than the additive effect of liraglutide alone plus dapagliflozin alone. It is worth highlighting that this was particularly unusual in sequential, as compared to simultaneous initiation of therapy [6]. In addition, the long duration of diabetes and insulin treatment in Patient #1 were actually predictors of poorer glycemic response to high-dose liraglutide [17]. Conversely, the high baseline HbA1c and long duration of liraglutide treatment in Patient #1 were predictors of greater HbA1c reduction with high-dose liraglutide [17]. 

In addition, the improvement in glycemic control was disproportionately greater than would be expected from the weight loss, with a loss of just 1.5% of weight at 12 weeks. The actual degree of weight loss was likely masked in the initial phase by the catabolic effect of uncontrolled hyperglycemia. In support of this, she went on to lose 6.3 kg (6.4% of weight) at 49 weeks, which was similar to the mean weight loss of 6.4 kg at 56 weeks in the SCALE Diabetes study [12]. While on treatment with high-dose liraglutide, loss of weight of ≥4% at 16 weeks was predictive of ≥5% weight loss at 56 weeks [12]. Thus, it has been proposed that if patients do not achieve ≥4% weight loss at 16 weeks, the physician should consider stopping liraglutide and/or switching to an alternative pharmacological agent for treatment of obesity [18]. If this recommendation was applied to Patient #1, liraglutide might be prematurely terminated. Thus, when initiating liraglutide, it is important to consider the baseline degree of glycemic control and recognize that early weight loss might be masked in patients with uncontrolled hyperglycemia. 

In Patient #2, the magnitude of this weight loss was unusual and usually associated with bariatric surgery—certainly, it was much higher than in the aforementioned studies on combination therapy in which weight loss of 2.8–3.3 kg was observed [7,9,10]; as well as the SCALE Diabetes study in which high-dose liraglutide led to an approximate weight loss of 3% in 6 weeks [12]. Clearly, the weight loss response was far greater than the additive effect which might be anticipated from liraglutide or SGLT2 inhibitor monotherapy. Higher baseline weight, treatment with metformin and a longer duration of liraglutide treatment predicted a better weight loss response to high-dose liraglutide. Of these, the first two factors were applicable to Patient #2.

Another observation worth highlighting in Patient #2 was the rapid weight loss over just 6 weeks, which was followed by a plateau in weight despite up-titration and continuation of high-dose liraglutide for 12 months. This was an unusual observation for two reasons—firstly, the rate of initial weight loss was exceedingly rapid, and secondly, this weight loss was achieved with the lowest dose of liraglutide (0.6 mg daily), seemingly defying the well-documented dose-dependent effect of liraglutide [12].

What could account for the discrepancy in the weight loss response between Patient #1 and #2? The first point to highlight is that both patients were females. Contributed by the dramatic decrease in estrogen following menopause, there is a shift in fat distribution such that the visceral fat deposit increases from 5–8% of total body fat during the pre-menopausal period to 15–20% of total body fat during the post-menopausal period [19]. GLP-1 agonists, including liraglutide, have been demonstrated to decrease visceral fat and increase subcutaneous fat via tissue-specific modulation of lipid metabolism, as well as stimulate browning of subcutaneous white adipose tissue [20]. Therefore, this could explain the significantly greater weight loss in Patient #2 who was post-menopausal, compared to Patient #1 who was pre-menopausal. Conversely, what could account for the discrepancy in glycemic control between Patient #1 and #2? Estrogen leads to improved insulin sensitivity and glucose homeostasis—therefore, the combined effect of GLP-1 agonists and estrogen may explain the greater improvement in glycemic control observed in Patient #1 who was pre-menopausal compared to Patient #2 who was post-menopausal [21]. Putting all of this together, this could also account for the disproportionately greater improvement in glycemic control compared to weight loss in Patient #1, as earlier discussed. 

Are there any differences in the response to GLP-1 agonists between males and females? In a retrospective study of patients with Type 2 DM treated with exenatide for 1 year, males had improved glycemic response while females had improved weight loss response [22]. In support of the latter observation, females displayed higher sensitivity to the food reward impact of central GLP-1 receptor activation, postulated to be estrogen-mediated, in an animal study [23]. However, the former observation is at odds with the previously described effect of estrogen on improved insulin sensitivity, and could be explained by other factors such as the duration of DM and treatment with other medications for DM. Apart from this study, there has been a stark paucity of data in this area. Certainly, future large-scale studies are required to further study the effect of gender and menopausal status on the clinical response to GLP-1 agonists.

In a real-world study of patients with Type 2 DM on liraglutide, maximum decrease in HbA1c and weight was achieved after 12 months and 18 months respectively [15]. Thus, why did an early plateau of the effect of liraglutide occur in Patient #2? Relevant to this observation was a study performed by Farr et al. [24], in which participants who received liraglutide with dose escalation over 5 weeks had increased activity over the reward-related right orbitofrontal cortex (OFC) on functional MRI (fMRI) in response to food cues when controlled for body weight and BMI [24]. This counter-regulatory increase in OFC activity could explain the weight plateau in Patient #2, and is supported by other studies in which the early decrease in reward-related central nervous system activation in response to food cues after 10–17 days of liraglutide treatment subsequently disappeared after 12 weeks [23]. This suggests that apart from an “early responder” group of patients, there might also be an “early plateauer” group, in whom we could consider down-titrating liraglutide to the minimum dose in which therapeutic benefit can be achieved to reduce adverse effects and save costs. On the opposite end of the spectrum are patients with minimal response to therapy. Thus, it is evident that individual response to high-dose liraglutide is highly variable. A postulated etiology is genetic polymorphisms of the GLP1 receptor and related genes—a promising field deserving of further research [25].

## 4. Conclusions

In conclusion, I wish to summarize the key learning points from both cases as follows:

(1) Due to complementary mechanisms of action, combination therapy with high-dose liraglutide and SGLT2 inhibitor has significant clinical benefits including the potential for marked improvement in glycemic control and weight loss.
(2) Initiation of combination therapy can be sequential or simultaneous. In clinical practice, sequential initiation is usually the preferred approach and is not inferior to simultaneous initiation in the long term.
(3) Individual response to combination therapy with high-dose liraglutide and SGLT2 inhibitor is highly variable. Although the response to liraglutide is typically dose-dependent, a subset of patients might have an exquisite response to low doses.
(4) High baseline HbA1c and longer duration of treatment predict increased HbA1c reduction, while high baseline weight, treatment with metformin and a longer duration of treatment predict increased weight reduction.
(5) Clinicians can use ≥4% weight loss at 16 weeks as a marker of response to treatment, although an important caveat is patients with poorly controlled DM who might have an apparent absence of initial weight loss.
(6) A plateau in clinical effect is typically observed after 12–18 months, although it might occur as early as 6 weeks after therapy initiation.

## Data Availability

Not applicable.
